# A telemetry study shows that an endangered nocturnal avian species roosts in extremely dry habitats to avoid predation

**DOI:** 10.1038/s41598-023-38981-2

**Published:** 2023-07-23

**Authors:** Yohay Wasserlauf, Ady Gancz, Amir Ben Dov, Ron Efrat, Nir Sapir, Roi Dor, Orr Spiegel

**Affiliations:** 1 School of Zoology, Faculty of Life Sciences, Tel Aviv University, 6997801 Tel-Aviv, Israel; 2 The Exotic Clinic, 9978600 Gezer, Israel; 3 Yitshaki 11, Petah-Tikva, Israel; 4 Mitrani Department of Desert Ecology, Jacob Blaustein Institutes for Desert Research, Ben-Gurion University of the Negev, 8499000 Midreshet Ben-Gurion, Israel; 5 Department of Evolutionary and Environmental Biology and Institute of Evolution, University of Haifa, Haifa, Israel; 6 Department of Natural Sciences, The Open University of Israel, Ra’anana, Israel

**Keywords:** Behavioural ecology, Biodiversity, Climate-change ecology, Conservation biology, Ecosystem ecology

## Abstract

Describing animal space use is essential for understanding their ecological needs and for planning effective conservation schemes. Notably, certain biomes and life histories are understudied due to methodological challenges in tracking animals in their natural habitats. Specifically, both arid environments and nocturnal species are not sufficiently researched compared to diurnal species and to other biomes. This knowledge gap hinders our ability to properly prioritize habitats for species protection in areas undergoing human-related development. Here, we investigate the movement ecology of the Egyptian Nightjar (*Caprimulgus aegyptius*) in the arid Dead-sea region of Israel, the Palestinian Authority (the West Bank) and Jordan. This nocturnal insectivore is a cryptic desert-dweller and was considered locally extinct until it was rediscovered in 2016. For this work we tracked twelve individuals using GPS tags to determine how this resource-poor environment affects their home range, (predicting large areas), habitat use, and day-roost ecology. We found that the tracked Egyptian Nightjars had a much larger home range area than other Nightjar species, commuting nightly between foraging grounds and day-roosts. We found, as expected, intensive foraging activity at agricultural fields, where artificial irrigation likely supports higher resource (insect) density. Additionally, we found that individuals showed very high roosting site fidelity, often returning to the same specific site, located in extremely dry and exposed habitats, presumably for predator avoidance. This finding highlights the ecological value of these barren habitats that are often considered “lifeless” and therefore of lower priority for conservation. Consequently, our research demonstrates the importance of describing the space-use of nocturnal animals in arid habitats for conservation efforts.

## Introduction

Basic knowledge and familiarity with animal species is required for adopting suitable conservation measures to protect wildlife and biodiversity. Studying features such as: diet, natural history, space use patterns, behavior, small- and large-scale movements are often the first step before planning and conducting conservation actions.

The nocturnal niche represents half of the time on this planet. Yet, we often focus our research on diurnal species or limit our studies to periods of diurnal activity, and leave the nocturnal niche under-represented in ecological scientific research^1,2^. Ignoring or underestimating the importance of the nocturnal niche might hide the true importance of certain habitats, or the true nature of nocturnal or partially nocturnal species, especially in a mostly diurnal class such as Aves. This is particularly important when considering the ongoing wildlife and biodiversity loss due to human activity^3,4^. For instance, rice fields were identified as important habitat and stop-over sites for migratory birds in East Asia^5^. This finding provides ecological motivation to conserve rice fields, and to allocate resources to protect this kind of habitat, as well as take it into account in broad conservation plans. But for many species, we still do not know basic aspects of movement ecology such as home range (HR) size and habitat use (i.e. their movement ecology)^6,7^, despite their importance in determining where and how to maintain our protected areas^8,9^.

Arid ecosystems are characterized by higher levels of evapotranspiration, resulting in relatively poor productivity and biodiversity in comparison to water-rich biomes^10^. Hence, desert-dwelling species often avoid the intense daytime heat by being nocturnal, and tend to forage across larger home ranges to fulfill their needs^11–13^. These natural history traits and other biases result in the much larger representation in ecological studies of tropic and temperate climates and their endemic species compared with desert habitats and their species^14,15^. Thus, information about the movement ecology of nocturnal desert species, is a long-standing knowledge gap hampering effective conservation of these unique ecosystems and species^16^. Characterization of roost/nest areas and foraging grounds is a key component of species movement ecology, especially if individuals show high fidelity (loyalty) to specific sites, eventually facilitating more effective local species conservation^17–19^. Roosting and nesting areas may differ from foraging grounds, which might require different properties to be chosen by a specific species. Roosting and nesting habitats usually require relative safety both from disturbances such as harsh weather, predation risk, and competition (both interspecific and intraspecific) while foraging areas first need to fulfill feeding needs as well as ideally being free from the above-mentioned disturbances. For example, nesting sites can be limited spatially in the case of cliffs for various raptors, and cavities for some cavity nesters. These varying foraging and roosting needs may force species to commute between rich foraging patches and suitable roosting and nesting sites within their HR. For instance, Tristram’s grackles (*Onychognathus tristramii*) in the arid Dead Sea region commute between roosts at preferred rocky areas and foraging areas near anthropogenic (irrigated) green areas^20^. Similarly, insectivores in arid regions often focus their foraging activity around human-dominated habitats (settlements and agriculture) as these tend to be rich in water, productivity, and resources^21^.

The nightjar (*Caprimulgidae*) family consists of small to medium-sized, nocturnal, aerial-insectivores with a total of 97 species living in diverse biomes^22^. A few family members from temperate climates, such as the European Nightjar (*Caprimulgus europaeus*) and the Common Nighthawk (*Chordeiles minor*) have been intensively studied (e.g.^23–26^). In contrast, very little study, if any, has been done on the other species, particularly desert species which are hypothesized to differ in their space use patterns by using larger HR due to the paucity of resources in the desert environment. Hence, the habitat use and movement ecology of desert nightjars remains poorly studied, with potential implications for their conservation. Specifically, understanding the influence of settlement and agricultural developments on the nightjars’ behavior is crucial for effective conservation. In addition, greater understanding of desert nightjar movement ecology could provide insights applicable to the broader avifauna of deserts and other insectivores.

To begin to redress the lack of research on the space use patterns of nocturnal desert species, and the influence of human habitats, we focused on the Egyptian Nightjar (*Caprimulgus aegyptius*), a nocturnal insectivore, which breeds in the West Bank, in the extremely arid desert at the northern shores of the Dead-Sea. The Egyptian nightjar was considered a locally extinct breeder in Israel and in the West Bank since the 1940’s, only to be re-discovered in 2016 breeding in a previously unfamiliar breeding area for the species (see details below and in^27^). To the best of our knowledge, no previous studies directly addressed questions related to the species’ movement ecology or space use. This knowledge gap together with this species small population size in Israel and the West Bank, make it a high conservation priority nationally^28^. Also, the case of the Egyptian Nightjars can be seen as a model for other nocturnal insectivores of arid areas. Accordingly, we aimed to (1) estimate its HR size and spatial location, (2) identify habitats used for foraging, roosting, and nesting, and (3) test if individuals repeatedly used the same sites, which would make these sites critical for population monitoring and for the protection of the population and the unique habitat it inhabits. Towards this end, we tracked free-ranging Egyptian Nightjars and analyzed their HR size, movements, and habitat usage throughout the night, as well as their consistency in day-roosting locations. We hypothesized that in contrast to other nightjars from temperate zones, Egyptian Nightjars will forage at fertile oases and agricultural fields and commute to their day-roosts with some level of individual consistency^12,20,21^.

## Methods and materials

### Study species

The Egyptian Nightjar (*Caprimulgus aegyptius* *-* Lichtenstein 1823) is a small-sized (~70–80 grams), nocturnal insectivore, feeding mostly on moths and alike, that it catches on the wing^29^. Like many other Nightjar species, Egyptian Nightjar relies on their camouflage for protection, nest on the ground and lay 2 eggs in a small and exposed depression in ground^30,31^. The species is globally classified as "Least Concern" by the IUCN (International Union for Conservation of Nature), and it has a wide, but disconnected known global distribution range, from Morocco in the west, to Kazakhstan and Pakistan in the east. It inhabits a variety of desert habitats, including semi-desert areas and desert steppes, usually with scattered vegetation^29^. Two subspecies are mentioned in the literature: *C. a. saharae* is the western (African) subspecies, and *C. a. aegyptius*, is the eastern (Asian) subspecies. While some populations are known as migratory, others are known as resident. The species has been found in aggregated group roosts, with a record of up to 40 birds together in the United Arab Emirates^32^.

In Israel and in the West Bank, the Egyptian Nightjar was a rare breeder in sandy areas along the Mediterranean coastal plain until the first half of the 20th century. Since the local extinction as a breeder, the species remained only as a rare migratory bird^27,33^. In the summer of 2016, a new population was found at the northern part of the Dead Sea region in an area that was not known as a potential breeding habitat for the species^27^. Indeed, a few nests were found in the following years, about 70 years after the last confirmed breeding in the coastal plain in Israel. Due to the estimated small population size and its locality, and because data on the population size and extent are crucially needed for undertaking conservations actions, the Egyptian Nightjar was classified as “critically endangered” in Israel and in the West Bank, with a presumed migratory breeding population^27,28^.

### Research area

**Figure 1:**
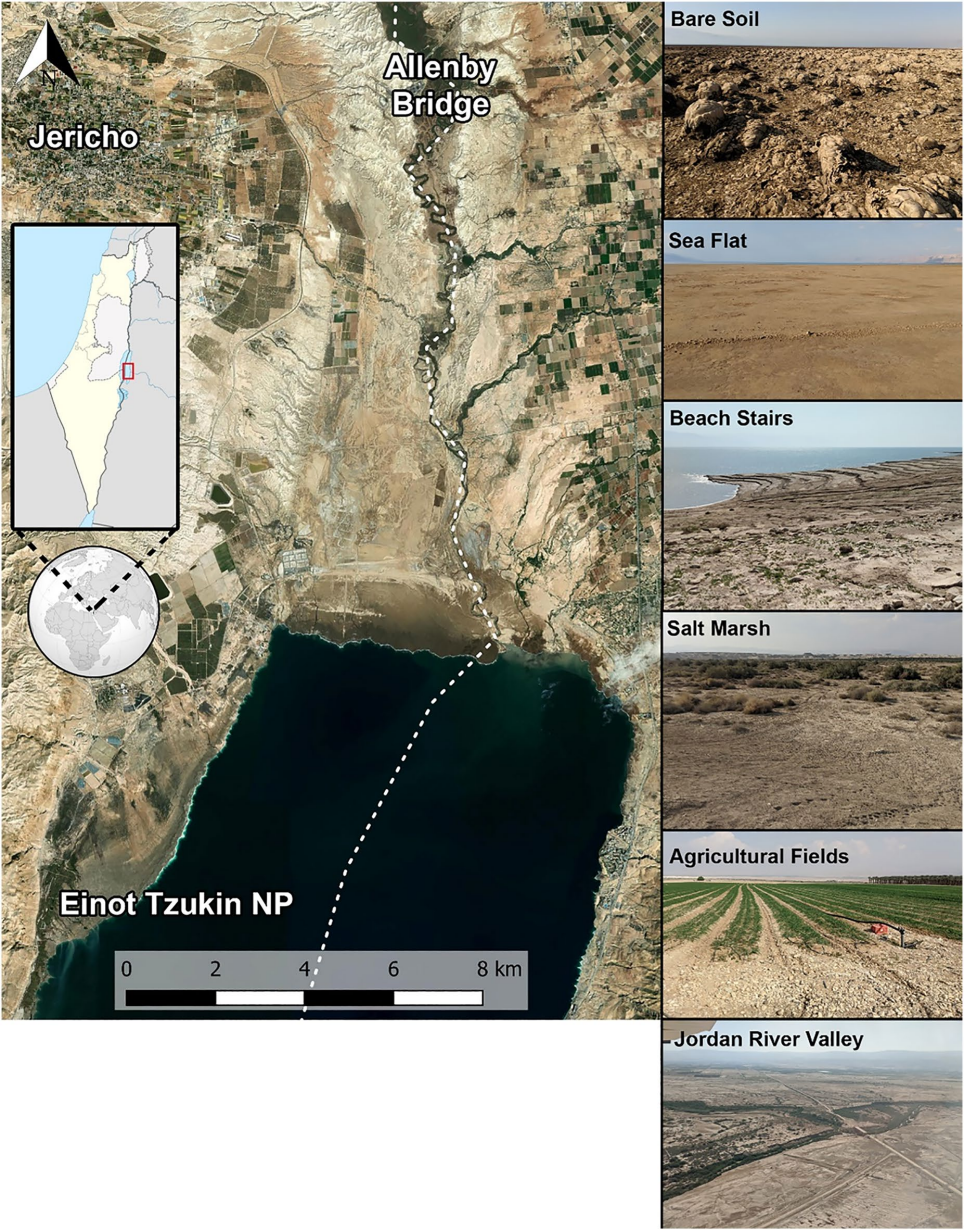
General map of the research area and examples for the different habitat types in the region. The dotted white line marks the border between the West Bank and Jordan. The map was created using QGIS 3.4.8 Madeira (https://qgis.org/en/site) & Adobe Photoshop CS6 (https://www.adobe.com/products/photoshop.html), Photos by Yohay Wasserlauf. Small maps by Wikipedia, under CC BY-SA 4.0.

This research focused on the breeding population of Egyptian Nightjars in the northern part of the Dead Sea region between Einot Tzukim Nature Reserve in the south, and Allenby Bridge in the North (Fig. 1). The Dead Sea region is known as the lowest place in the world, with a minimum elevation of 420 m below sea level. It is an extreme rain-shadow desert characterized by a very hot and dry climate, especially during the summer when temperatures rise above 40 °C for most of the day, often for several months continuously. During winter, on cold nights the temperatures can drop below 10 °C, and although rain and flashfloods do occur, annual precipitation is as low as 94 mm, resulting in extreme aridity (Data from the Israeli Meteorological Center).

The combination of the vegetated and non-vegetated habitats, partially created by the continuous drying and retraction of the Dead Sea over the last few decades, has created a unique landscape with diverse desert habitats. Richer habitats include several freshwater springs and the Jordan River, as well as irrigated agricultural fields and date (*Phoenix dactylifera*) plantations. On the drier end of the spectrum are completely barren habitats (without a single plant in sight; Fig. 1), reflecting a mixture of dry and high salinity soils such as salt marshes, stony plains and creeks, and sandy erosion flats, including "Beach stairs" along the shores of the Dead Sea that are created because of its retraction. These habitats are arranged in a compact mosaic, allowing traversing multiple habitats within a few kilometers (See appendix S1). Presumably, this mixture supports the local population of the Egyptian Nightjars, utilizing the different habitats according to their changing needs across diurnal and seasonal cycles.

The Jordan River runs from north to south, separating the Kingdom of Jordan to the east and Israel and the West Bank to the west. The area is sparsely populated with only one big city (Jericho, never visited by our tracked Nightjars), a few small settlements and tiny army bases scattered throughout. Around 20,000 people are estimated to live in the area, mostly in Jericho (data from Palestinian Central Bureau of Statistics and Megilot regional council Website). Along the international border there is a 1–3 km wide strip, which is a closed military zone with no access to civilians and thus limited human activity.

### Tagging and movement data

We captured Nightjars using standard mist-nets during dusk and dawn, and throughout the night during the spring (March–June) of 2019 and 2020, at their roosting and feeding sites, using playbacks to attract the birds. Once captured, standard ringing protocols included metal ring attachment, wing and tail length measurement (using 1 mm resolution ruler) and weighing (to the nearest 0.1 g). We determined age and sex by feather molt stage and morphological characters^29,30,32^. We also sampled blood from the brachial vein (up to 50 µL) for genetic analysis, and for sexing verification^34^. For tracking the movements of Egyptian Nightjars, we fitted the birds with GPS tags. We attached PinPoint GPS-VHF 50 (N=5, 2019) and PinPoint GPS-VHF 75 (N=9, 2020; both manufactured by Lotek Wireless Inc.) using a 3 mm Teflon strap in a backpack configuration. The devices weighed 3.0 g and 3.5 g respectively, adding less than 5% to the bird’s body mass. Tags were set to record the location of the bird every hour during the night, and once during the day (at noon). Birds tagged in 2019 were recorded hourly from 19:00 to 05:00 and birds tagged in 2020 were recorded hourly from 18:00 to 06:00 (Israel Standard Time, GMT+2), allowing us approximately up to two months of tag battery life. The data collected by the tags was downloaded remotely from up to 2 km, using a dedicated antenna and a base station unit (Lotek Wireless Inc.).

### Ethics statement

This study was conducted in compliance with ARRIVE (Animal Research: Reporting of In Vivo Experiments) guidelines, and all capturing, ringing, and tagging procedures were done following the guidelines and regulations set by the Israeli Nature & Parks Authority (INPA), under birds ringing license No. A287 and research permit No. 2019/42175, both issued by the INPA. Additionally, Close supervision and field attendance by the INPA rangers was provided during the fieldwork in this study, to ensure the ethical standards of this project. The study, and all the bird handling and tagging protocols and precedes were also approved by the ethical committee of Tel Aviv University.

### Data processing and statistical analysis

#### Data preparations

First, we filtered out poor-quality GPS fixes. We excluded fixes obtained with fewer than 5 satellites, or with high (>4) HDOP (Horizontal Dilution of Precision – (see^35^ for detailed information))^36^. Second, we differentiated nocturnal locations from diurnal locations, classifying each location as "Night" or "Day" according to the sun’s position above or below the horizon. Third, to account for the seasonal changes in night duration, which may potentially affect Nightjars activity timing, we calculated a uniform "night progress” measurement. This index ranges from 0% (sunset, the beginning of the night) to 100% (sunrise, the end of the night) with some margins before and after sunset and sunrise (e.g. 110% is around an hour after sunrise, and similarly, − 10% is around an hour before sunset), so an hour is represented by a percentage value, depending on the seasonal variation in night duration. We processed and analyzed the data using R software (R Core Team 2016).

#### Home range analysis

We calculated the Kernel Density Estimations (KDE) for 95% (hereafter "home range", HR) & 50% ("core area") of the utilization distribution of each bird, based only on the nocturnal locations across the entire track. We used the R package "adehabitatHR" and HREF calculation^37^ as an ad hoc method to create KDE polygons and identical grids for all individuals. Since HR sizes depend on tracking duration that varied among individuals, we also calculated the "home range" and "core area" of the first 20 tracking nights for birds that were tracked for at least 20 days. According to our sensitivity analysis, in our particular dataset this threshold minimized data loss while ensuring the generality and quality of the estimates: HR growth was asymptotic after this period, resulting in reliable estimates of the final HR size across the full tracking period, allowing the inclusion of the majority (~61%) of the tracked Nightjars (excluding 2 juveniles, see Appendix S2 and S3 for home range and core area accumulation curves as a function of tracking duration for both adults and juveniles). To support the hypothesis that desert species occupy larger HRs, we compared our results with those available from the literature for European Nightjars^38,39^.

#### Habitat usage analysis

To understand the Egyptian Nightjars' habitat usage and how it changes throughout the night, we calculated the birds' percentage of presence in all the habitats they were recorded in. First, we mapped habitat polygons with aerial photos and ground truthing survey (on the accessible west side of the border), classifying areas into one of the habitats, from resource-rich to resource-poor, as described in Fig. 1 (See appendix S1). Then, we classified each of the recorded locations to their polygon/habitat and calculated the percentage of presence for each of the habitats, for each hour, throughout the night and during the day for all the individuals together. To investigate the circadian changes in the Nightjars’ activity we compared individual-specific day (roost) and night (foraging and commuting) points in a paired design. In addition, using the R package "lme4"^40^ we modeled the use of agricultural fields (presumably the main foraging habitat) with a linear mixed model as a function of nightly progress and individual ID as a random factor.

#### Nocturnal movement pattern analysis

We used two indices to investigate the nightly movement pattern of tracked individuals: ‘Displacement’ – defined as the bee-line distance from preceding roost point to each nocturnal location. Here we excluded two juveniles from the analysis (the only juveniles in the data) as they vary substantially in their behavior from adults, presumably due to exploration behavior. ’Hourly step size’- defined as distance between every pair of consecutive points throughout the night, describing the Nightjars short term movements.

#### Day roosts analysis

To understand the ecology behind the Nightjars' diurnal roost use, and the tendency of individuals to return to the same location, we analyzed the daily return rate. We calculated the distance between consecutive day-roost locations (first and last points of each night) and aggregated these as percentage of the nights (from the total number of nights) that ended at three range-classes from their start point: The same exact spot- within 20 m (accounting for possible GPS errors); The same general location (within 100 m); and the same broad area (within 500 m). Here we included only birds with 10 or more daily estimates (N = 11).

## Results

**Figure 2:**
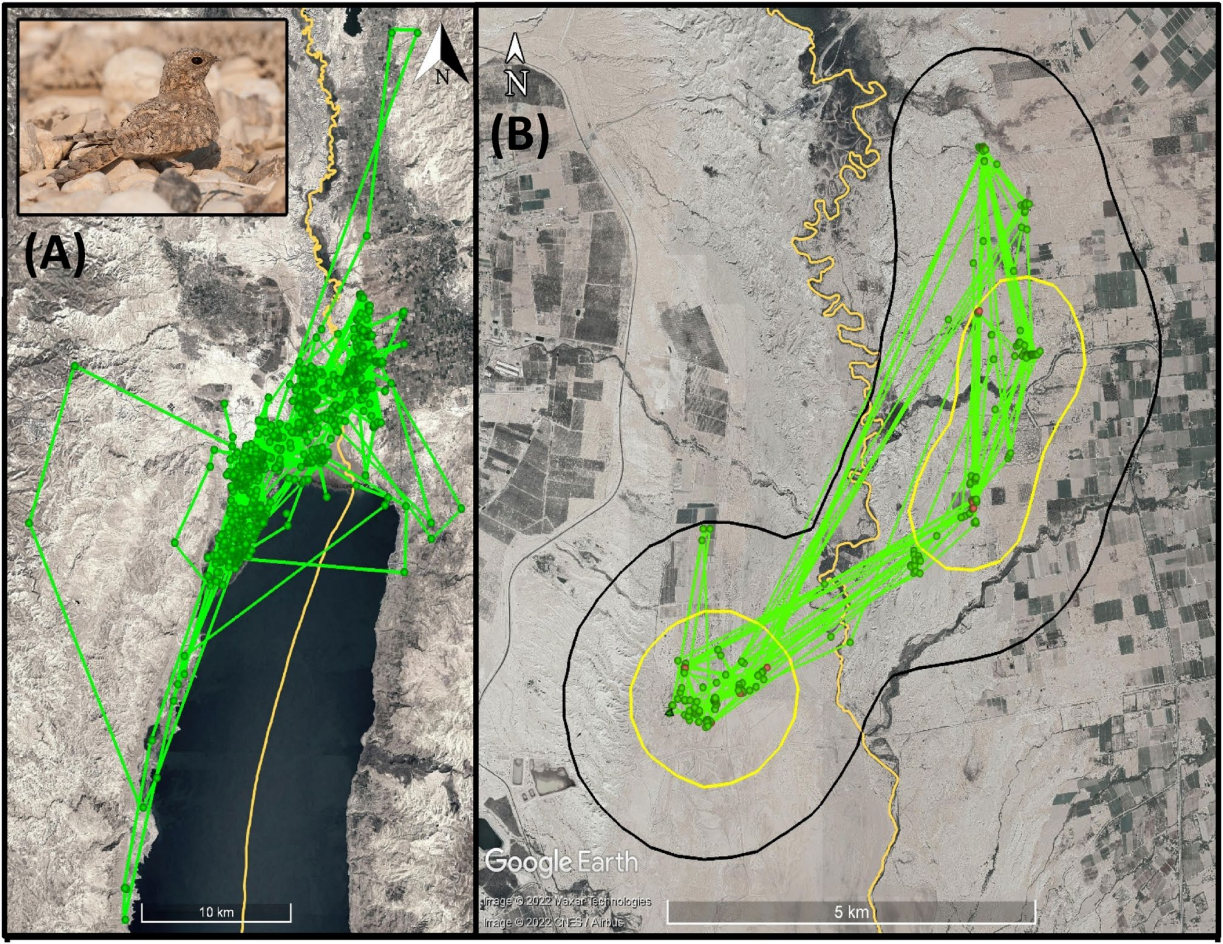
(**A**) A general overview of the combined GPS tracks for all the Nightjars that were recorded during the study. The inset shows an adult Egyptian Nightjar (Caprimulgus aegyptius). Green dots represent a recorded location, green line represents a track between two consecutive points. (**B**) An example of a home range map of one of the tracked Nightjars, the black line represents the 95% KDE area, and the yellow lines represent the 50% KDE area. The orange line marks the border between the West Bank and Jordan. This individual was active on both sides of the border, mostly within the closed military zone along the border. The map was created using Google Earth Pro 7.3.6 (https://www.google.com/earth/versions) & Adobe Photoshop CS6 (https://www.adobe.com/products/photoshop.html), Egyptian Nightjar photo by Yohay Wasserlauf.

We deployed GPS tags on 13 Egyptian Nightjars during two field seasons. We were able to download the data from 12 of them with a tracking duration of 28.1±17.7 days (mean±SD, range: 8 - 68; Table 1), retrieving a total of 4895 bird locations, out of 5079, before filtering (Fig. 2). Five (3 adults and 2 first-year juveniles) and 8 (all of them are adults - second year or above) birds were tagged during 2019 and 2020, respectively. This count includes one of the birds that was tagged in 2019 and was recaptured and retagged in 2020, and due to the different life stages of the bird it was analyzed as two different individuals in some of the analyses. 9 males and 4 females are included in the dataset. Broadly speaking, individuals completely avoided date plantations, tended to day-roost in the driest habitats and foraged in the agricultural fields, up to 4 km from their respective roosts.

**Table 1:** A summary of the Egyptian Nightjar tracking dataset.

**#**	Bird ID	Tracking year	Sex	Age	Tracking duration (d)	GPS locations	Core area	Overall HR	Core area (20 days)	Overall HR (20 days)
1	tag_1914F	2019	Female	Adult	10	97	1.1	8.9		
2	tag_1926M	2019	Male	Adult	18	192	3.4	16.0		
3	tag_1936M	2019	Male	Adult	21	233	1.1	8.0	1.1	8.0
4	tag_1943M	2019	Male	Juvenile	47	533	4.0	34.7	9.0	48.9
5	tag_1953M	2019	Male	Juvenile	60	687	6.4	51.5		
6	tag_2016M	2020	Male	Adult	68	827	3.4	20.0	3.7	23.9
7	tag_2025F	2020	Female	Adult	30	405	9.0	37.0	6.7	30.0
8	tag_2036F	2020	Female	Adult	8	109	3.5	24.1		
9	tag_2055F	2020	Female	Adult	13	177	1.8	9.2		
(5)	tag_2065M (=1953M)	2020	Male	Adult	27	367	1.8	11.5	2.0	11.0
10	tag_2075M	2020	Male	Adult	46	619	4.6	22.5	7.7	33.2
11	tag_2085M	2020	Male	Adult	21	273	4.5	20.1	4.5	19.9
12	tag_2096M	2020	Male	Adult	28	376	1.3	15.3	1.4	14.2
	Mean ± SD				28.1±17.7	350.7±220.3	3.3±2.2	19.0±9.6	4.6±3.0	23.6±13.5

Home ranges (HRs) were estimated using Kernel density estimator (KDE). To account for variation in tracking duration when comparing individuals, HR core area (KDE 50%) and overall HR size (KDE 95%) were calculated both for the entire tracking duration, and (if applicable) also after 20 days of tracking.

### Home range (HR) analysis

After 20 days of tracking, we found that Egyptian Nightjars have home and core range sizes of 23.6±13.5 km^2^ and 4.6±3.0 km^2^ for KDE 95% and KDE 50%, respectively (mean±SD, N= 12). HR sizes for the entire (individual-specific) tracking period were 19.0±9.6 km^2^ for KDE 95%, and 3.3±2.2 km^2^ for KDE 50%. Notably, these values are about 10 times larger than the values reported by Evens et al.^38^ for European Nightjars in temperate environments, with a reported HR of 0.44 km^2^ for KDE 50%, and 1.9 km^2^ for KDE 95% (t=8.38, df=58, *p*<0.0001). Similarly, they are also larger than the overwintering HR (0.05 km^2^ for 95% KDE) reported for Eastern Whip‐Poor-Will (*Antrostomus vociferus*) by^41^, also in a more temperate environment (t=5.77, df=22, *p*<0.0001). Unfortunately, our sample size is too small to identify known sources of intra-specific variation in HR size (e.g. age, sex and personality^42^).

### Habitat usage

**Figure 3:**
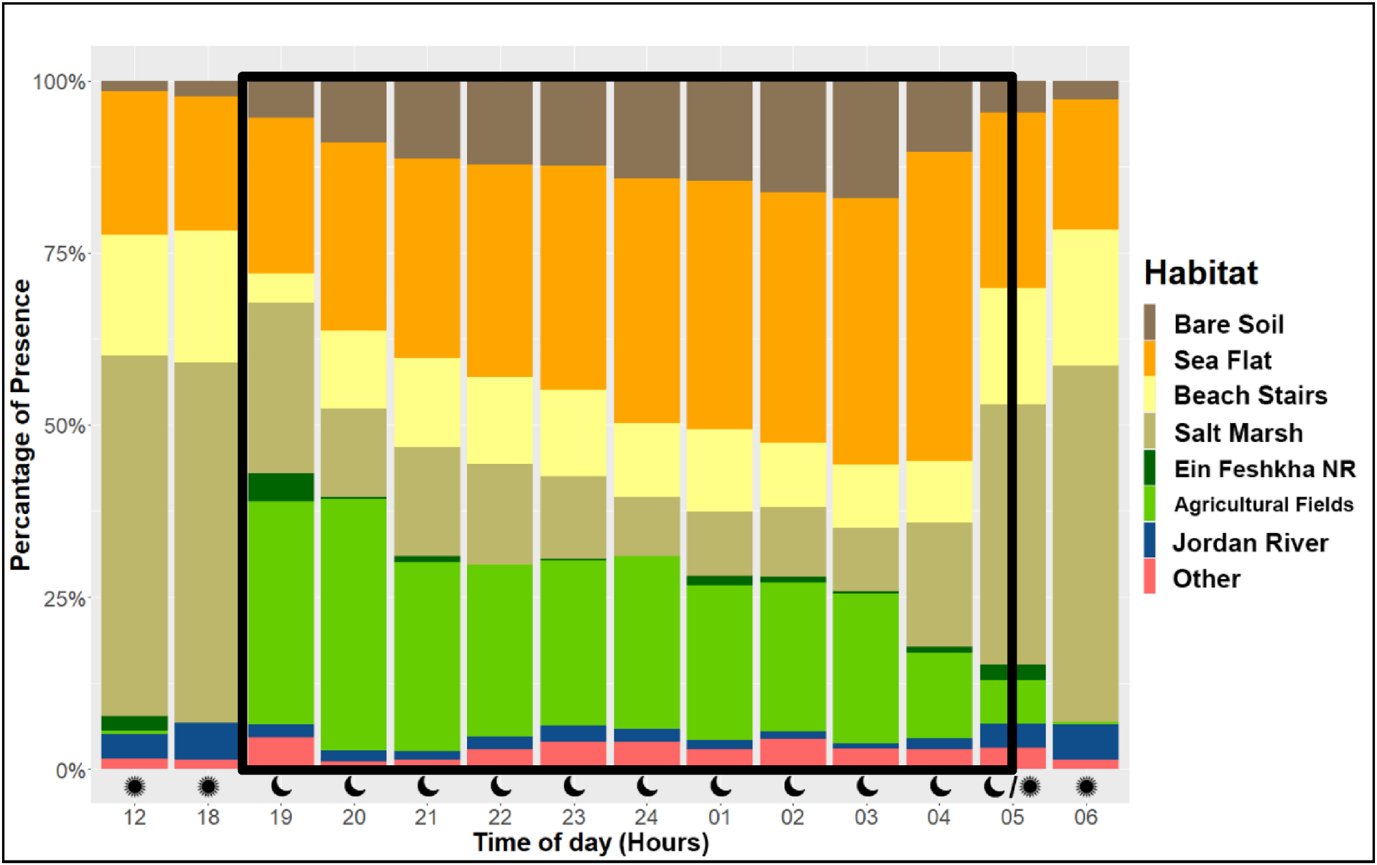
Average habitat use for all tracked birds during the night (black frame: from 19:00 to 04:00 or 05:00 depending on the season) and the day (12:00, 18:00, 06:00 and 05:00 according to the season). The X-axis represents the time of day, and the Y-axis represents the cumulative percentage of habitat use (18:00 and 06:00 records were only available for birds that were tagged in 2020). Day roosts were almost exclusively in dry habitats (particularly salt marshes), while nightly activity was centered around agricultural fields, with a diminishing trend during the night.

To investigate the Egyptian Nightjars’ habitat usage throughout the night and during the day, we calculated their presence at each habitat. First, our results show a difference between nocturnal habitat usage and diurnal habitat usage. During the day (mid-day, or first/last point of the night, before/after departure) the Nightjars were found significantly more in drier and exposed habitats such as Sea Flats, "Beach Stairs" and salt marshes, compared with more wet areas such as agricultural fields and the Jordan River (Paired Wilcoxon test: V=2813, *p*<0.001; Fig. 3). Second, during the night we found high rates of presence in agricultural fields, especially earlier during the night, in addition to presence in the above-mentioned dry habitats. The presence rates in agricultural fields and other vegetated habitats decreased during the night (model results: intercept: 40.03, Night progress effect: -0.368). This finding indicates a gradual shift to more exposed-dry habitats towards the end of the night (Fig. 3). Notably, although date plantations are the most common agricultural cultivation in the north Dead Sea region (Megilot Regional Council Website), Nightjar locations were never within this irrigated and potentially food-rich habitat type, suggesting a very strong avoidance by the Nightjars.

### Daily movement patterns

**Figure 4:**
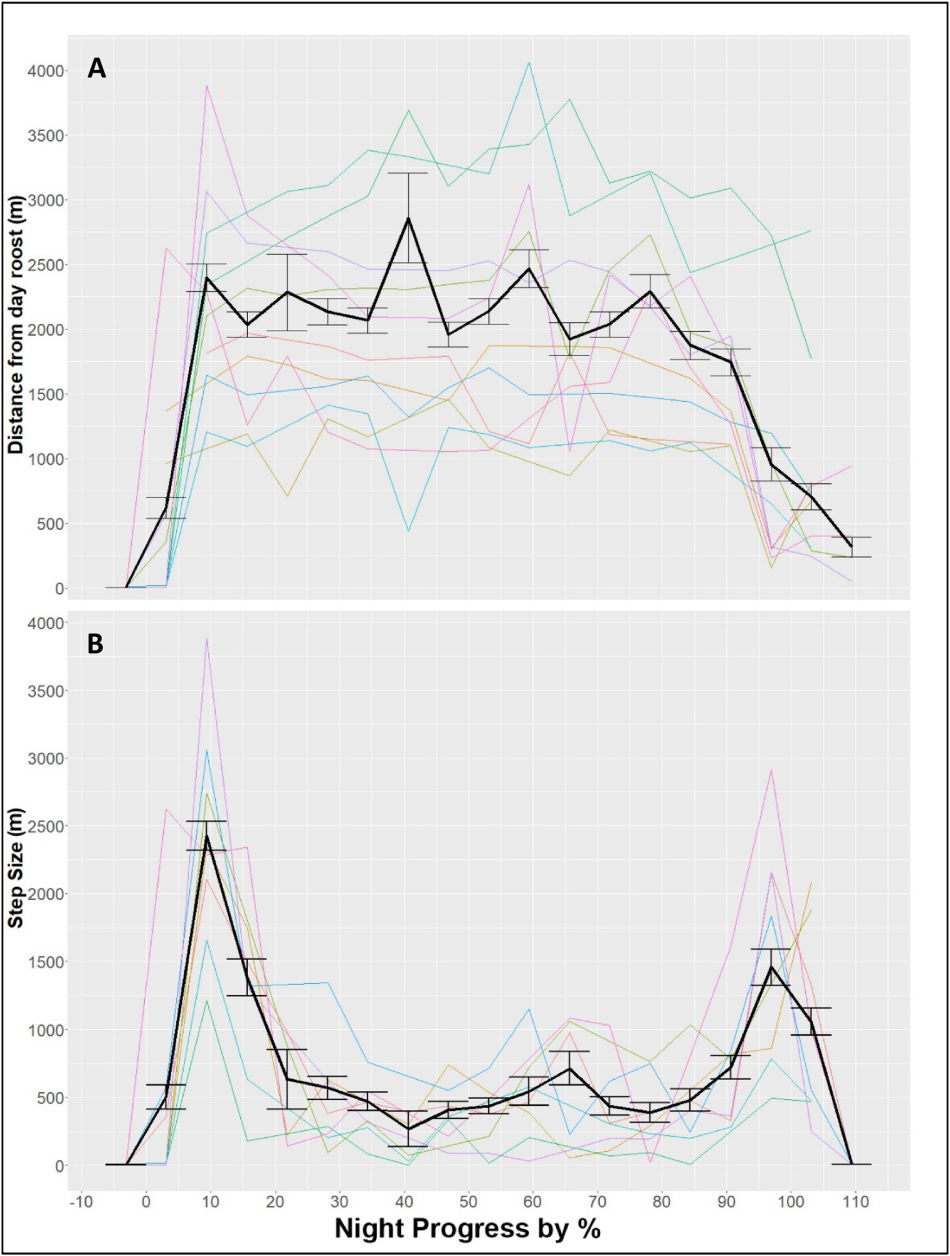
(**A**) Mean displacement from roost site for all adult birds, by night progress (0% - sun set, 100%- sunrise). (**B**) Mean step size values recorded for 2020 birds only, by night progress. Colored lines represent different individuals (averaged across all nights for each one, variation not shown, see appendix S4). The bold black line represents the general mean, with standard error bars among individual means.

We found strong spatial separation between nocturnal activity areas and diurnal roost sites. Foraging sites were usually 1–4 km away from the preceding or following roost sites (and individuals tended to return to the same day-roost site, see below). At the beginning of the night (within the first hour of leaving the roost), the Nightjars performed an extended commute flight to an average distance of about 1–3.5 km, reaching their foraging areas (Fig. 4A). Then, they spent most of the night in areas within a similar distance from the day roost site, gradually shifting habitats but not moving further away. At the end of the night, the nightjars performed a return commute flight that resulted in a sharp reduction in the displacement distance to the original day-roost. While initial commutes were synchronized among all individuals (at the first 10–20% of nightly progress), return commutes were far less so (spanning over 80–110% of nightly progress), and were often spread over a longer period - split into a few stops on the way back to the roost area at the end of the night (Fig. 4A).

The profile of step sizes during the night provides more support for this description. The two commutes are indicated by periods of two clear peaks of longer steps at the beginning (~2400 m during the first hour - ~15% of night progress) and end of the night (~1500 m). During the middle part of the night, we found that on average, the Nightjars perform shorter steps, without long-distance flights (typically less than 500 m per hour, on average) which would probably be reflected even with only an hourly resolution. This suggests that they are staying at the same general area, or moving gradually to different foraging areas, away from their roost point.

On some nights, longer steps were occasionally recorded during the middle part of the night (see Appendix S4), reflecting movements between distant foraging habitats or a short movement toward the roost location. Such steps might explain the shorter step sizes on the return commute at the end of the night (compared to the outbound commute). This high variation of step length during the middle part of the night, with a lack of synchrony across the different nights and individuals, results in shorter mean step sizes, compared with the beginning and end of the night (Fig. 4B).

### Day roost ecology

**Figure 5:**
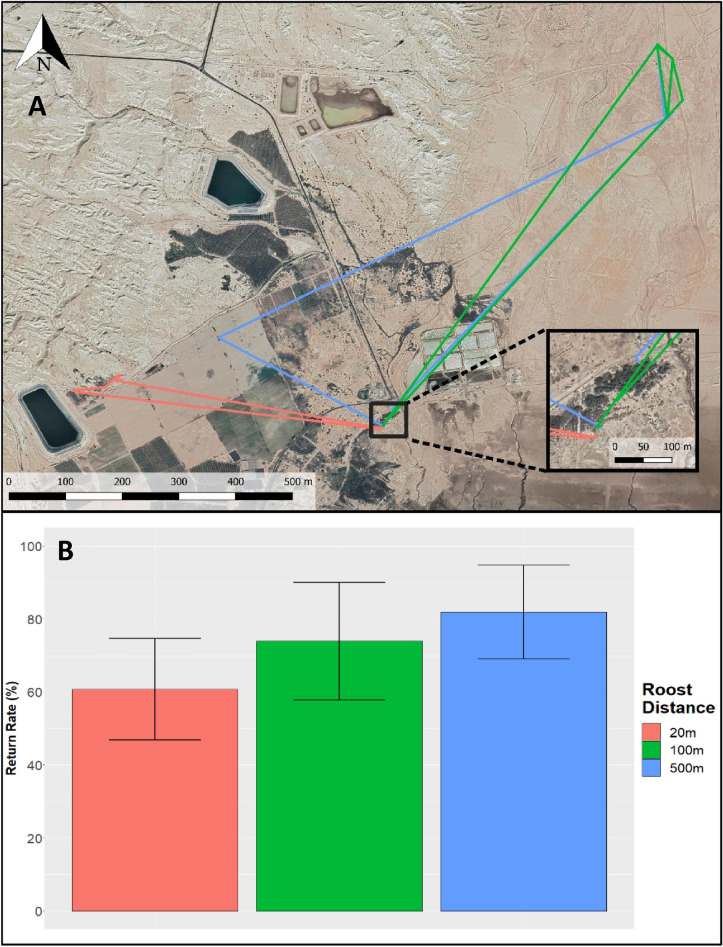
(**A**) Example map of 3 nights from one individual, each night ended at a different distance from where it started: red<20 m, green<100 m and blue<500m. (**B**) Weighted average (±SD) for return rates to diurnal roosts for all tagged Nightjars with more than 10 days of tracking. Each color represents the rate of nights that ended at a certain distance (in meters) from the night starting point. The map was created using QGIS 3.4.8 Madeira (https://qgis.org/en/site) & Adobe Photoshop CS6 (https://www.adobe.com/products/photoshop.html).

We analyzed the return rates to the same roost locations on successive nights. Our dataset included a total of 326 days with consecutive roosts from 11 individuals (after the exclusion of individuals with less than 10 days), with a mean of 29.64 nights ±15.36 (mean±SD). We found that most of the nightjars (60.7%±13.89 weighted average for all tagged birds, range: 40–85% per bird) were found less than 20 m from the point where they started, which indicates very high nightly return rate (Fig. 5A). When extending the spatial distance between the beginning and the end of the night to 100 m and 500 m we found weighted average nightly return rates of around 74%±16.12 and 82%±12.90, respectively, which also support a strong return rate to the same roosting area (average and weighted average led to similar results).

## Discussion

In this study, we used GPS tags to track and analyze the nocturnal movement of Egyptian Nightjars, a desert insectivore from a recently discovered population with high national conservation priority. In agreement with our hypothesis for desert insectivores, we found that individuals had relatively larger home-ranges and core-areas in comparison to other Nightjar species. We also found that Egyptian Nightjars routinely utilize a diversity of habitats. After commuting for 1–4 km from their day roost, they typically started their nightly foraging in food richer areas such as agricultural fields (but not date plantations). Then they gradually shifted to the drier and more exposed areas, eventually spending the day roosting in extremely barren habitats. This transition during the night, as well as the daytime habitat use, and back-and-forth commutes might be explained by predator avoidance behavior with a shift to predator-free habitats once foraging is completed. This is due to the ground-roosting behavior of the Egyptian Nightjars and the relative high presence of predators in the richer areas, compared to the dry-exposed areas. We also found that individual Egyptian Nightjars show a strong reliance on specific roosting points (often shared sites), with a high return rate to the same point for many consecutive days. Overall, these findings provide important insights into the Egyptian Nightjars ecology, allowing the application of conservation measures. Specifically, they highlight the importance of specific roost sites within extremely exposed habitats for this population - areas that are typically overlooked as potential candidates for conservation because they hold very low biodiversity and virtually no productivity. Below we discuss the HR of this Nightjar species concerning other species, its habitat uses and movement ecology, its day roosting behavior and finally the conservation implications of our findings.

### Home-range and core-area sizes

The home-range estimates found in our study (of ~21 km^2^ and ~3 km^2^ for the entire HR and core area, respectively; Table 1) are considerably larger than other Nightjars species. These HRs are about 10-fold larger than those found for both European Nightjars in Belgium (~2 km^2^ and 0.4 km^2^ for HR and core areas^38^) and for the Nubian Nightjar (*Caprimulgus nubicus*) in southern Israel (~1 km^2^ for KDE 95%^43^). However, in these two studies the tracking was done using standard radio-telemetry (with “pinging” tags), which might affect the home-range size estimation because this method is sensitive to long and remote flights, potentially biasing towards smaller estimates^44^. Nevertheless, much smaller HRs (~ 0.05 km^2^) were also reported in GPS-based study of the wintering areas of Eastern Whip‐Poor‐Will in Central America^41^. More broadly, our HR size estimates are larger than those expected from allometric scaling relationship for birds of this size, especially given the short tracking duration (~20 days)^45–47^. Although HR size calculations might be affected by methodological differences (e.g. tracking duration, sampling design or analysis protocols), the big dissimilarities suggest that these results represent real biological differences among the three species and their respective environments (i.e. higher mobility and lower resource availability, respectively).

Two plausible explanations for our findings are the low resource density in such an extreme arid area, in comparison to other biomes (e.g. temperate Belgium or tropical Central America), and a predator avoidance strategy. Interestingly, smaller home ranges were documented for desert-dwelling Nubian Nightjar. This anomaly might reflect the fact that the Nubian nightjar is a specialist of salt marshes with tamarisk bushes, and used this resource both as day roosts and foraging grounds. This species is also much smaller than the Egyptian Nightjar (~25% less body mass), and therefore it might be satisfied even with the limited resources available within the close vicinity of its roosting site. Beyond limited resources, the larger home-range of the Egyptian Nightjars likely reflects a behavioral strategy and emerges from their tendency to commute to remote day-roosts in the most extreme exposed non-vegetated desert areas (Fig. 4). These long commutes between habitats fulfilling the different needs of the birds at different times contribute to the very large HRs of the species and reflect a trade-off between safety and resource richness, as we discuss below.

### Habitat usage and nocturnal movement pattern

We found a very clear geographical and habitat separation between the diurnal roost locations and the nocturnal activity areas of Egyptian Nightjars, as well as a shift in the habitats used during the night (Fig. 3). We explain this movement from food-rich to drier habitats as time minimization of presence in risky areas. Once foraging needs are satisfied, the Nightjars appeared to avoid the food-rich patches (e.g. agricultural fields and the areas around them) that also attract predators, and move to safer habitats such as dry and exposed areas, where predators are rare. While we did not directly quantify the presence of mammalian predators (mostly foxes and jackals in our system), or actual predation pressure in the different habitats, regular encounters during fieldwork support our argument that predator activity shows a strong positive gradient with local productivity. In contrast to the food-richer habitats, the extreme bare habitats, such as salt pans and recently exposed Dead Sea floor are too salty for any vegetation. These extremely barren conditions, where the only shade offered is by random and rare log sediments, show rare activity by other wildlife. Thus, there is an absence of mammalian predators and reduced predation risk in these areas.

The trend towards drier habitats with the progression of the night, also corresponds with our finding that Egyptian Nightjars spent the majority of the night at a distance of 1-4 km from their roost location (Fig. 4A,B). Such a separation has never been shown for any Nightjar species in arid habitats (likely reflecting mostly data deficiency in these regions rather than the rarity of the behavior). Yet, similar habitat separation was reported for the European Nightjar in temperate-forested habitats and for the Red-Necked Nightjar in milder climate and less forested habitat^23,38,48–50^. Those studies showed differences between the nesting/roosting habitats (roosting can be on trees) and the foraging ones, but over a small scale of a few hundred meters, and not the large scale of kilometers we found here. As discussed above, the greater spatial separation in our system may reflect an interaction of the habitat structure with the trade-off between safety and foraging for the Egyptian Nightjars. It is possible that the large recorded flying distances are caused by the man-made agricultural fields, which might encourage the Nightjars to fly further away from their roosting point for better foraging areas.

Finally, date plantations (palm trees) are a major agricultural industry in the area, covering vast areas, and rapidly spreading (Megilot Regional Council Website). They are irrigated and thus are presumably richer patches than the surrounding desert, especially during the dry periods covered in our tracking. Nevertheless, none of the focal Egyptian Nightjars entered a date plantation, indicating a strong (and somehow counterintuitive) avoidance. We speculate that as a visually oriented aerial insectivores, Egyptian Nightjars are challenged by the hunting conditions inside the plantations relative to open habitats. Whether for this reason or another, the clear avoidance pattern implies that when planning new dates plantations or expansion of the ones already existing, it is important to take into account the drastic effect of this type of agriculture on the Egyptian Nightjars behavior and possibly also its population persistence, in order to avoid a potential land-use conflict in the region.

### Day roost ecology

In addition to their nocturnal behavior, the Nightjars’ diurnal roost ecology is important for their natural history as well as for identifying points of high conservation priority. Like other Nightjars species, the Egyptian Nightjars usually roost in the shade of a small bush (if available) during the day, relying on their effective camouflage and their thermoregulation abilities to remain unnoticed^51–53^. We found that they show high levels of roost site fidelity with very high return rates to the same roost area. On average, the majority (~60%) of all nights ended at the same point of departure in the beginning of the night, and ~80% of the nights ended at the same area where they started (Fig. 5). High fidelity to specific roost sites was found for other species with similar ecology or occupying similar habitats. In Canada, Common Nighthawks maintain the same roost tree and even return to the same branch^54^, and the very limited wintering range recorded for each individual Eastern Whip‐Poor‐Wills (spanning merely over a few hundreds of meters), might reflect the same roosting place during the whole winter^41^. These high-fidelity values highlight the importance of day roost locations for the conservation of nocturnal or desert species in general and for the Egyptian Nightjars in particular.

During the research period we managed to find 7 Egyptian Nightjars’ nests (one of them of a tagged bird), all of them in the same areas and apparent habitat as those used for roosting. While these instances are insufficient for spatial statistical analyses, this agreement between nesting and roosting sites implies that preserving day roost areas and specific sites can potentially contribute directly for preserving nesting areas and is, therefore, crucial for the conservation of the locally endangered Egyptian Nightjars in the region. Generally, most of the roost sites were in dry, sometimes non-vegetated areas. While investigating the characteristics making specific spots preferred over other similar looking places nearby is beyond our scope here (and no obvious trait was apparent from our anecdotal observations), given the high fidelity observed this is definitely a possible key avenue for future studies of this species and others.

## Concluding remarks and conservation implications

Nocturnal species and those active in arid areas present a knowledge gap in various aspects of their life history, precluding their effective conservation. By harnessing advanced technology with miniaturized GPS tags, we were able to investigate several aspects of the movement ecology of the locally endangered Egyptian Nightjar with straightforward implications for their conservation, and for this generally understudied pristine environment.

First, Egyptian Nightjars’ relatively large HRs demonstrates that establishment of large, protected areas in arid habitats is essential. In Israel and in the West Bank, many of the arid areas are included in massive nature reserves^55^, but globally, arid habitats are under-represented in protected areas^15^. Second, tracking data can further facilitate mapping of key habitats for the Nightjars’ various activities. Overall, we found that the Egyptian Nightjars use a "mosaic" of habitats with foraging areas and roosting areas away from each other, and conserving the Egyptian Nightjars population requires the protection of the full "mosaic" pattern with sufficient distance between the different habitats. This is needed to create gaps between areas where predators are present and areas where predators are absent. Third, their intensive use of extremely barren habitats within this arid area highlights the importance of inclusion of these areas, usually presumed to be biologically unimportant. In the case of the northern Dead-Sea region, we refuted the assumption that there is no "life" in those areas and established their critical importance for the species roosting and nesting phases and hence for the persistence of the population as a whole. Presumably, the lack of predators in these habitats might also be an attractive factor to other wildlife species with strong movement abilities, especially nocturnal species, that may use them as shelters from predators. Fourth, major areas around the northern shores of the Dead Sea are restricted military zones without public access due to their proximity to the border. Regardless of other implications of these closures, these turn out to be beneficial for Egyptian Nightjars, especially as day roost areas. Nevertheless, other areas frequently used by Egyptian Nightjars are currently unprotected by any law and they possibly face future development. In particular, we find that date plantations, although common (and spreading) in the region are avoided by Nightjars completely (all activities included). Therefore, when expanding these monocultures, we should consider their negative impact on desert dwelling species in general, and specifically ensure that enough natural habitat is left as a buffer around agricultural fields to allow Nightjars to reach potential roosts with a feasible commute distance.

This fascinating, small and recently discovered population of Egyptian Nightjars, and the species as a whole has many more secrets and undescribed traits that can help us understand how birds and other animals can survive in such extreme conditions, such as those characterizing the Dead Sea area, and how they cope, both physically and behaviorally with such a habitat. The conservation of nocturnal species is sometimes a challenging task, but it's our mission to study, conserve and protect those special species and their unique habitats for a better natural world around us.

## Supplementary Information

Supplementary Figures.

## Data Availability

The datasets generated during and/or analyzed during the current study are available from the corresponding author on reasonable request.
