# Supplementary material for: A telemetry study shows that an endangered nocturnal avian species roosts in extremely dry habitats to avoid predation
Source: Sci Rep. 2023 Jul 23;13:11888. doi: 10.1038/s41598-023-38981-2 (PMC10363541; doi:10.1038/s41598-023-38981-2)
Supplement: Supplementary file 1 — Supplementary Figures. [file 41598_2023_38981_MOESM1_ESM.docx]

**Submission to Scientific Reports – Supplementary Material:**

**Title:**

A telemetry study shows that an endangered nocturnal avian species roosts in extremely dry habitats to avoid predation

Authors:

Yohay Wasserlauf^1^*, Ady Gancz^2^, Amir Ben Dov^3^, Ron Efrat^4^, Nir Sapir^5^, Roi Dor^6^, Orr Spiegel^1^

^1^School of Zoology, Faculty of Life Sciences, Tel Aviv University, Israel

^2^The Exotic Clinic, Almagor 5, Tel-Aviv, Israel

^3^Yitshaki 11, Petah-Tikva, Israel

^4^Mitrani Department of Desert Ecology, Jacob Blaustein Institues for Desert Research, Ben-Gurion University of the Negev, Midreshet Ben-Gurion, Israel

^5^Department of Evolutionary and Environmental Biology and Institute of Evolution, University of Haifa, Haifa, Israel

^6^Department of Natural and Life Sciences, The Open University of Israel, Ra’anana, Israel

**Appendix:**

1. Different
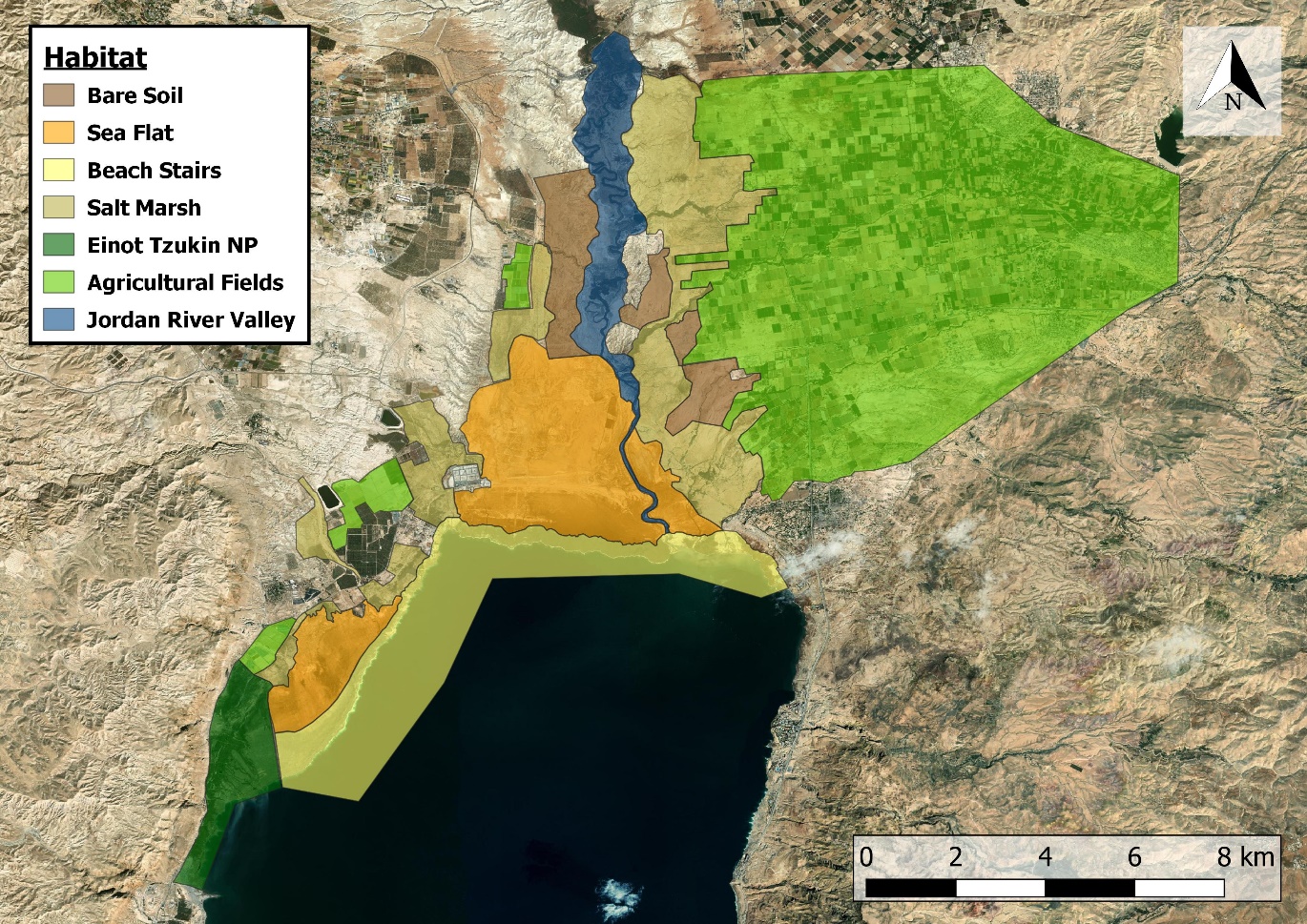
habitats in the research area (See Figure 1 for representative images):

Figure S1:

- **Agricultural fields and Dates plantations** – Cultivated fields of seasonally fruits and vegetables, irrigated and maintained regularly.
- **Salt marshes** - Salty lands with scattered vegetation of mostly bushes (mainly Tamarisk bushes and *Atriplex halimus*) and small trees.
- **Inner Jorden River valley** - The lowest part of the area where the Jordan River is flowing, surrounded by thick vegetation of reed and tamarisk trees (marks the border between Israel and Jordan).
- "**Beach stairs**" - Sloppy area with "stairs" that were created by the retraction of the sea, it is an extremely dry and almost completely non-vegetated habitat.
- **Sea flat** - An extremely dry and completely bare (non-vegetated) plateaus created by the retraction of the sea.
- **Bare soil** - areas of exposed dry lands prior to the sea retraction, completely uncovered by vegetation and highly exposed to the desert sun.

The map was created using QGIS 3.4.8 Madeira (<https://qgis.org/en/site>)

1. Home range accumulation curves:


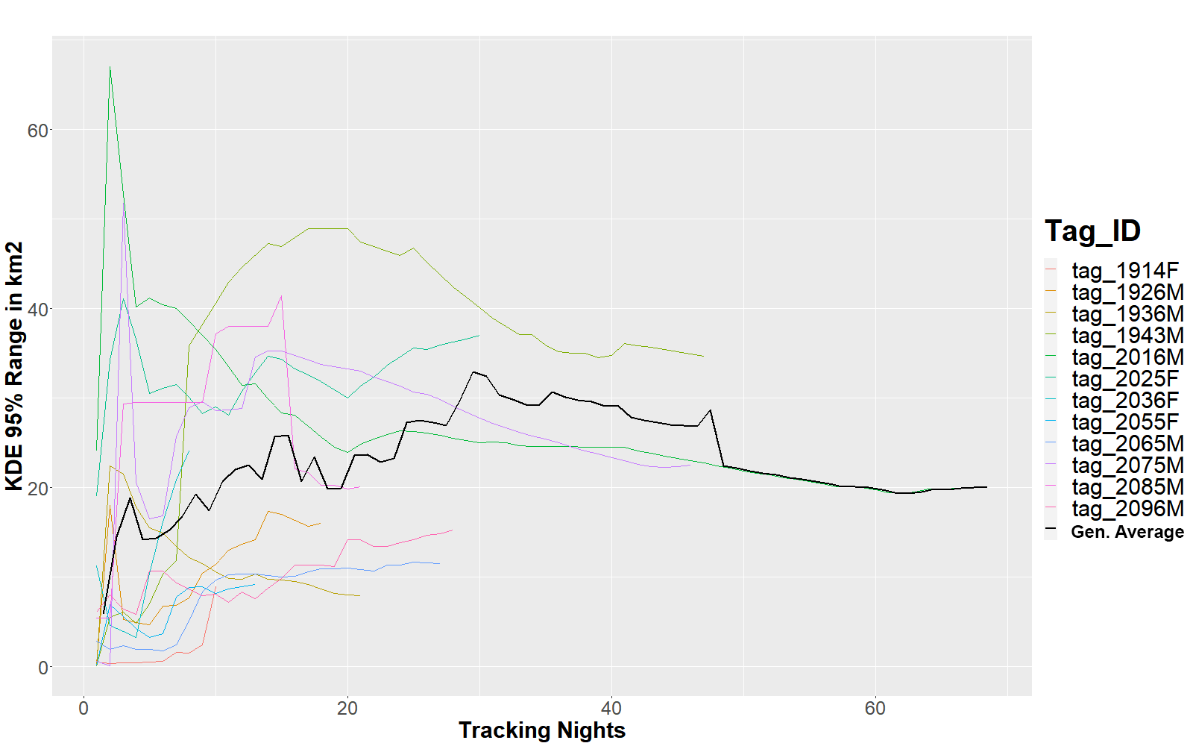


**A**


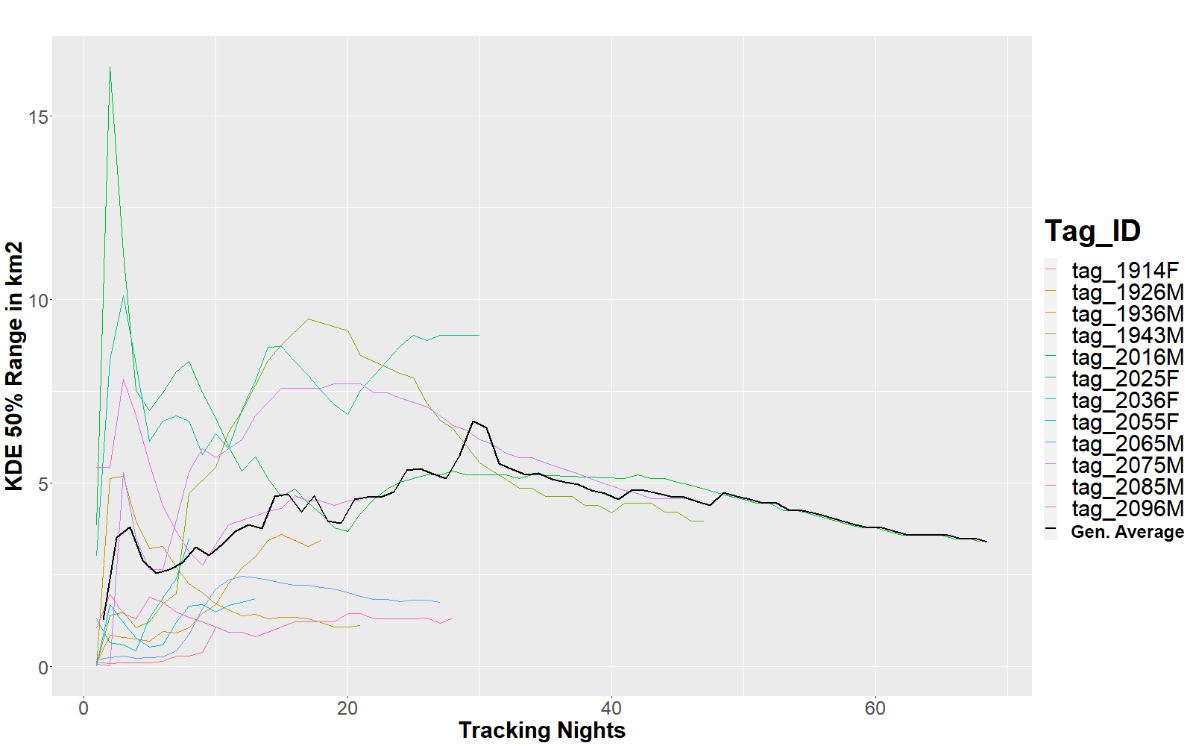


Figure S2: (A) – Home range (KDE 95%) size by tracking duration.
(B) – Core Area (KDE 50%) size by tracking duration.
The X-axis represents the tracking duration in nights, and the Y-axis represents the Home-range size in km^2^. Every color represents different individual, and the bold black line represent the general average. Blue dashed line marks 20 days.

**B**

1. Home range accumulation curves for juvenile birds:


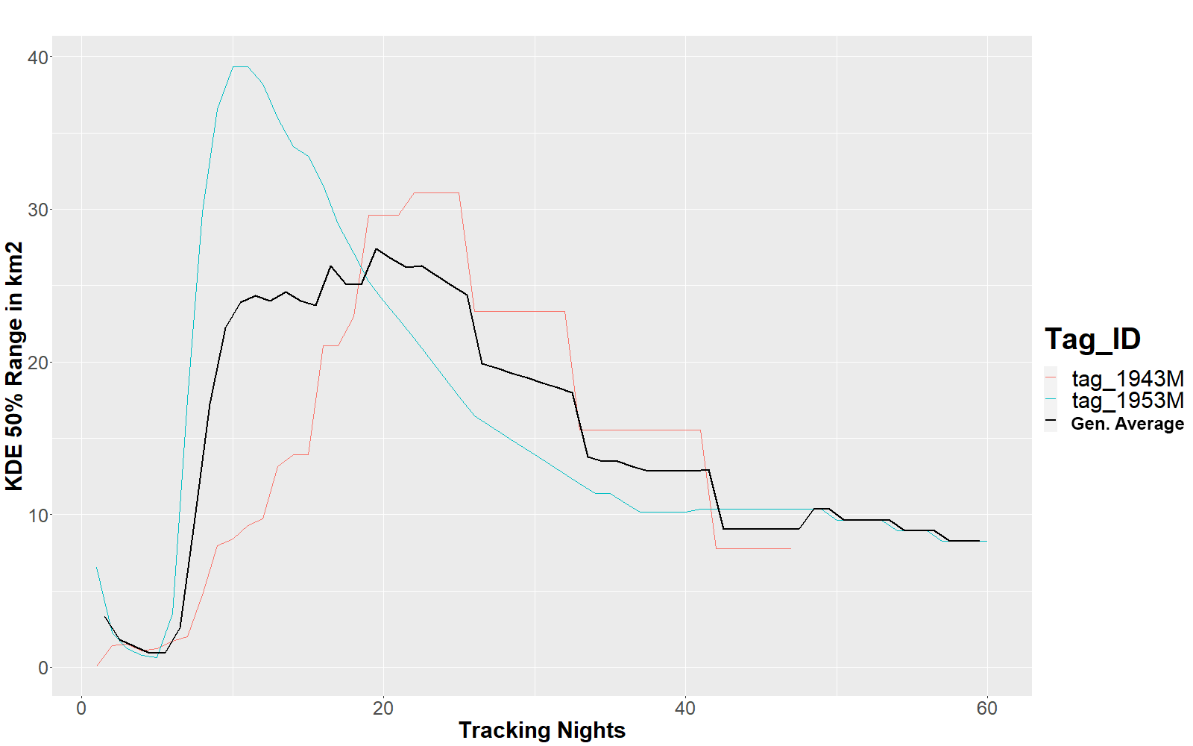

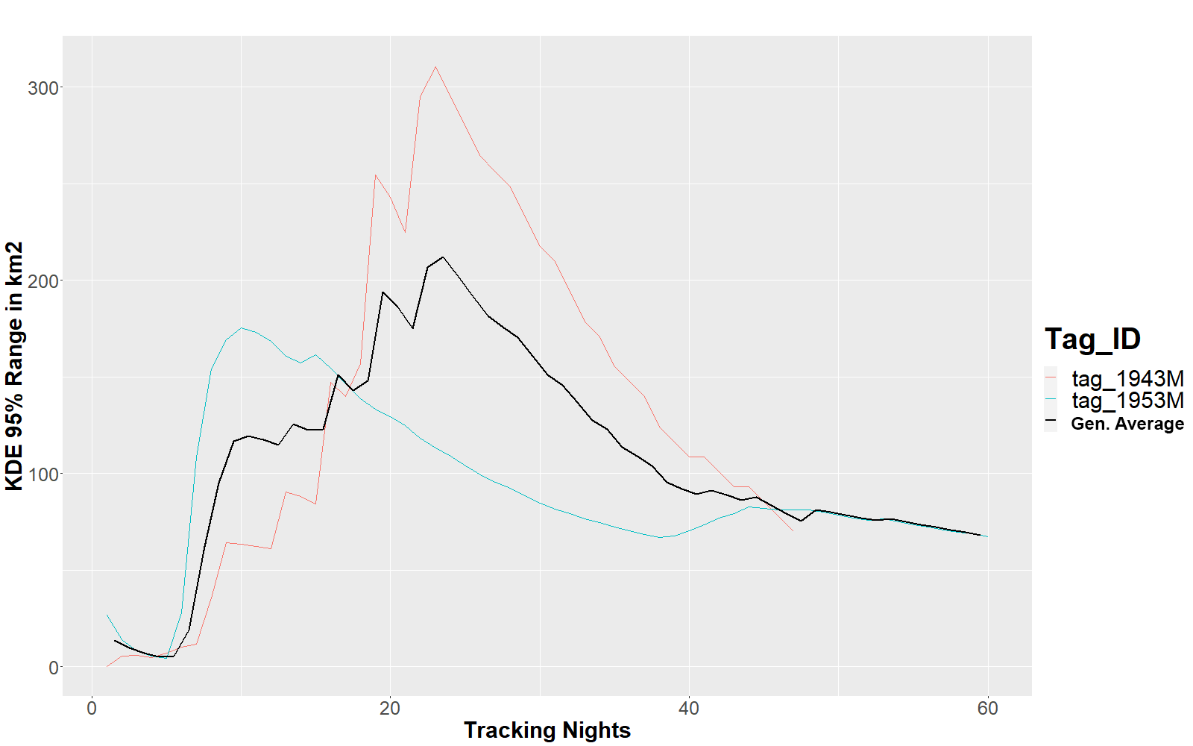


**B**

**A**

Figure S3: (A) – Home range (KDE 95%) size by tracking duration.
 (B) – Core Area (KDE 50%) size by tracking duration.
The X-axis represents the tracking duration in nights, and the Y-axis represents the Home range size in km^2^. Every color represents different individual, and the bold black line represent the general average. Blue dashed line marks 20 days.

1. Single night step sizes:


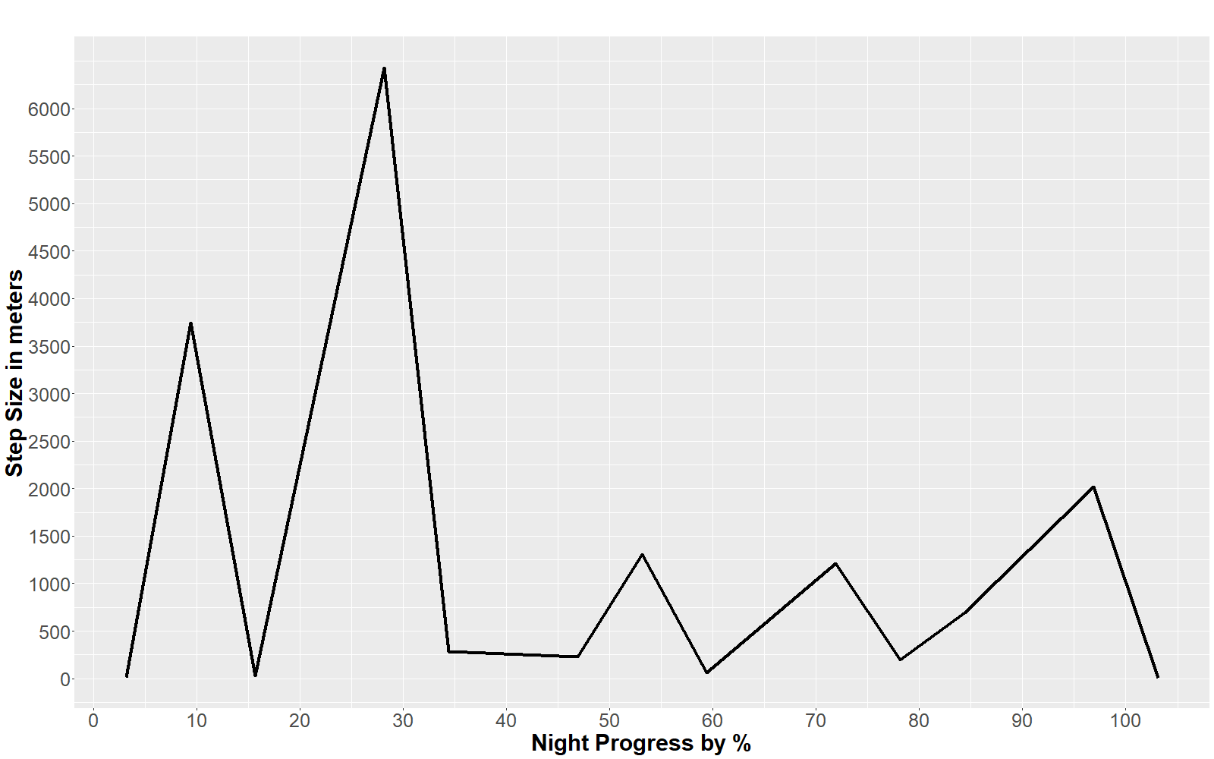


Figure S4: Example of step sizes for one individual, tag_2085M, representing longer step sizes during the night, without synchronization between them, resulting in lower means values.
